# Microalgal biofactories: a promising approach towards sustainable omega-3 fatty acid production

**DOI:** 10.1186/1475-2859-11-96

**Published:** 2012-07-25

**Authors:** T Catalina Adarme-Vega, David K Y Lim, Matthew Timmins, Felicitas Vernen, Yan Li, Peer M Schenk

**Affiliations:** 1 Algae Biotechnology Laboratory, School of Agriculture and Food Sciences, The University of Queensland, Brisbane, QLD 4072, Australia; 2 Centre for Metabolomics, School of Chemistry and Biochemistry, The University of Western Australia M313, 35 Stirling Highway, Crawley, WA 6009, Australia

**Keywords:** Docosahexaenoic acid, DHA, Eicosapentaenoic acid, EPA, Microalgae, Omega-3 fatty acids, Polyunsaturated fatty acids

## Abstract

Omega-3 fatty acids eicosapentaenoic acid (EPA) and docosahexaenoic acid (DHA) provide significant health benefits and this has led to an increased consumption as dietary supplements. Omega-3 fatty acids EPA and DHA are found in animals, transgenic plants, fungi and many microorganisms but are typically extracted from fatty fish, putting additional pressures on global fish stocks. As primary producers, many marine microalgae are rich in EPA (C20:5) and DHA (C22:6) and present a promising source of omega-3 fatty acids. Several heterotrophic microalgae have been used as biofactories for omega-3 fatty acids commercially, but a strong interest in autotrophic microalgae has emerged in recent years as microalgae are being developed as biofuel crops. This paper provides an overview of microalgal biotechnology and production platforms for the development of omega-3 fatty acids EPA and DHA. It refers to implications in current biotechnological uses of microalgae as aquaculture feed and future biofuel crops and explores potential applications of metabolic engineering and selective breeding to accumulate large amounts of omega-3 fatty acids in autotrophic microalgae.

## Introduction

Omega-3 (ω-3) fatty acids are polyunsaturated fatty acids (PUFAs) and essential components for the growth of higher eukaryotes [1]. Nutritionally, eicosapentaenoic acid (EPA, 20:5) and docosahexaenoic acid (DHA, 22:6) are the most important fatty acids belonging to this group of bioactive compounds. These long chain omega-3 fatty acids provide significant health benefits to the human population, particularly in reducing cardiac diseases such as arrhythmia, stroke and high blood pressure [2,3]. Additionally, they have been seen to offer beneficial effects to depression, rheumatoid arthritis and asthma [4-6].

Currently, the principal source of EPA and DHA for human consumption is marine fatty fish such as salmon, mullet and mackerel [7,8]. However, global catches have been in decline since the late 1980s and the number of overfished stocks has been increasing exponentially since the 1950s [9,10]. Furthermore, the presence of chemical contaminants (e.g. mercury) in fish oil can be harmful to consumers [11,12]. In addition, fish oil is not suitable for vegetarians and the odour makes it unattractive. There is a variety of alternative EPA and DHA sources such as bacteria, fungi, plants and microalgae that are currently being explored for commercial production. Fungi require an organic carbon source and typically long growth periods [13], plants need arable land, have longer growth times and have no enzymatic activity for producing long chain PUFAs EPA and DHA, unless genetically modified [14]. Microalgae are the initial EPA and DHA producers in the marine food chain and can naturally grow fast under a variety of autotrophic, mixotrophic and heterotrophic culture conditions with high long chain ω-3 fatty acid production potential [15]. Autotrophic and mixotrophic microalgae fix atmospheric carbon dioxide during photosynthesis, can potentially grow on non-arable land and have short harvesting times [16,17]. A comparison shows that microalgae can reach much higher EPA and DHA contents and productivities compared with other possible sources (Table 1). In particular heterotrophic microalgae are well established as an alternative source of DHA and are added to infant milk formula or other food [18]. Other microalgal products are used as food additives, animal feed (including aquaculture), vitamins, pigments, pharmaceutical compounds, cosmetics and potentially as a biofuel source [17,19,20]. The development of an efficient large-scale cultivation system for the commercial production of EPA and DHA would address a major global need. Here, we review the potential of autotrophic eukaryotic microalgae as biofactories for large-scale production of omega-3 fatty acids.

**Table 1:** Comparison of EPA and DHA fatty acid contents as percentage from total lipids in examples of bacteria, fungi, fish, transgenic plants and microalgae

**Organism**	**% EPA and/or DHA production**	**Reference**
Bacteria		
*Shewanella putrefaciens*	40.0 EPA	[21]
*Alteromonas putrefaciens*	24.0 EPA	[22]
*Pneumatophorus japonicus*	36.3 EPA	[23]
*Photobacterium*	4.6 EPA	[24]
Fungi		
*Thraustochytrium aureum*	62.9 EPA + DHA	[1]
*Mortierella*	20.0 EPA	[25]
*Mortierella*	13.0 EPA	[26]
*Pythium*	12.0 EPA	[27]
*Pythium irregulare*	8.2 EPA	[28]
Fish		
*Merluccius productus*	34.99 EPA + DHA	[29]
*Theragra chalcogramma*	41.35 EPA + DHA	[29]
*Hypomesus pretiosus*	33.61 EPA + DHA	[29]
*Sebastes pinniger*	29.8 EPA + DHA	[29]
*Oncorhynchus gorbusha*	27.5 EPA + DHA	[29]
*Mallotus villosus*	17.8 EPA + DHA	[29]
*Sardinops sagax*	44.08 EPA + DHA	[29]
*Clupea harenguspallasi*	17.32 EPA + DHA	[29]
Plant (transgenic)		
Soybean	20.0 EPA	[30]
*Brassica carinata*	25.0 EPA	[31]
*Nicotiana benthamiana*	26.0 EPA	[32]
Microalgae		
*Nannochloropsis* sp.	26.7 EPA + DHA	[33]
*Nannochloropsis oceanica*	23.4 EPA	[34]
*Nannochloropsis salina*	~28 EPA	[35]
*Pinguiococcus pyrenoidosus*	22.03 EPA + DHA	[36]
*Thraustochytrium* sp.	45.1 EPA + DHA	[37]
*Chlorella minutissima*	39.9 EPA	[38]
*Dunaliella salina*	21.4 EPA	[39]
*Pavlova viridis*	36.0 EPA + DHA	[40]
*Pavlova lutheri*	27.7 EPA + DHA	[41]
*Pavlova lutheri*	41.5 EPA + DHA	[42]
*Isocrysis galbana*	~28.0 EPA + DHA	[43]

## Microalgae in aquatic food chains: the initial omega-3 producers

**Figure 1:**
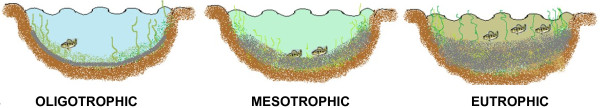
**Algal blooms in eutrophic aquatic systems use up nutrients and compete for light.** If nutrients become limiting first, microalgae may accumulate large amounts of lipids and/or carbohydrates as a survival strategy. The decay of organic matter by bacteria uses up oxygen causing localized anaerobiosis zones. These zones (here shown as grey areas) are present in all aquatic systems but occur at much deeper levels under mesotrophic or oligotrophic conditions. Photosynthetic microalgae require polar polyunsaturated lipids in particular for membrane where fluidity is critical, while most storage lipid occurs in the form of lipid bodies containing triacylglycerides. These typically vary in their composition and typically contain a mixture of saturated and unsaturated fatty acids for storage.

Microalgae are by far the most abundant primary producers that can be found in most aquatic systems, photosynthetically converting light energy and carbon dioxide (CO_2_) into biomass such as carbohydrates [44], proteins [45] and lipids [46]. Under high nutrient supply (eutrophic conditions), algae blooms commonly occur as microalgal cell density drastically increases [47]. During microalgal blooms the limitation of nutrients or light halters the increase of biomass. If nutrients, but not light, are limiting, this leads to the accumulation of photosynthetic bioproducts such as lipids and carbohydrates. These serve as storage products in order to survive the stressful growth limiting conditions, after which a large number of cells die [47,48]. Algal biomass is subsequently degraded by microorganisms, consuming large amounts of oxygen. As a result an anaerobic zone in the water is formed (Figure 1). In extreme cases, this can lead to anaerobiosis of the entire water body, causing the death of plants and animals in the waterway; interestingly this process is also believed to have been the key factor for large-scale oceanic anoxic events that led to fossil mineral oil deposition [17].

**Figure 2:**
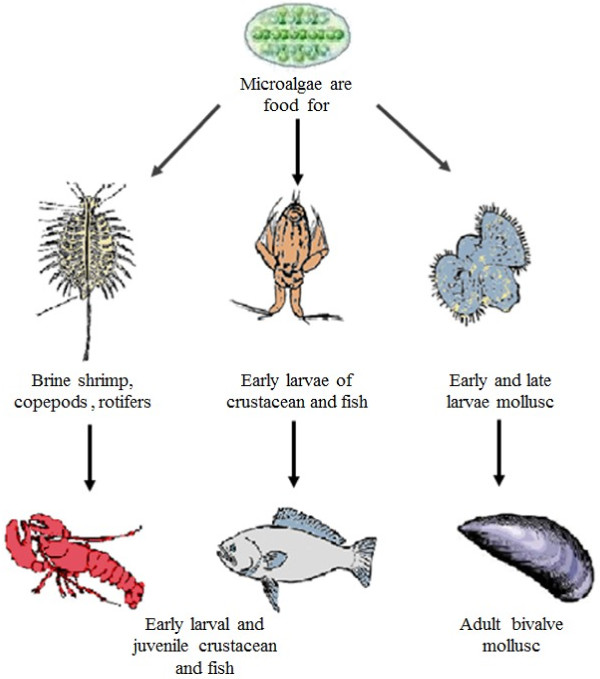
Microalgae are the primary food source of essentially all marine and freshwater food chains.

Importantly, microalgae are also the primary producers of EPA and DHA that are eventually accumulated through the various trophic levels. Changes in microalgal lipid content are carried on up the food chain (Figure 2), impacting the growth and dietary make-up of zooplankton, crustacean larvae, mollusc and some fish [49]. This subsequently affects the accumulation of EPA and DHA fatty acids in higher organisms and humans. Consequently, lipid profiles in microalgae play a vital role in maintaining the integrity of the world’s aquatic food webs.

## The nutritional importance of microalgae and EPA content in aquaculture

Microalgae are essential to the aquaculture industry which has grown substantially over the last 10 years [50,51]. The successful cultivation of oysters, scallops and mussels is dependent on the ω-3 fatty acids from microalgal feedstock. The polyunsaturated omega-3 fatty acids EPA and DHA derived from microalgae (e.g. *Isochrysis, Tetraselmis,Chaetoceros, Thalassiosira,Nannochloropsis*) are also known to be essential for healthy development of various bivalve larvae [52,53]. Prior research on the scallop *Pecten maximus* has shown a direct relationship between the fatty acid profile of female gonads and the fatty acid composition of the eggs [54]. The increase of EPA and DHA from an algal diet significantly increased the concentration of fatty acids in the digestive gland (78%) of scallops as well as the female (57%) and male gonads (51%). It appears that dietary lipids are stored in the digestive gland and are later transferred to the developing female gonad. These dietary lipids are then incorporated into the eggs and can significantly improve their quality. This in turn improves the hatching rate of eggs and hatching rates have been linked to high contents of EPA and DHA [53]. Aside from bivalve culture, microalgae are also used as food additives to improve the flesh color of salmon [55], as well as inducing a range of other biological activities such as survival and resistance [19].

The selection of suitable microalgae species for aquaculture is very important. Firstly, a candidate species must be adaptable to mass culture with high growth rates and lipid content [34,56]. Furthermore, it must tolerate moderate fluctuations of temperature, light and nutrients [57,58]. A microalgae species used for aquaculture must also have the appropriate size for ingestion (e.g. from 1 to 15 μm for filter feeders; 10 to 100 μm for grazers) and be readily digestible [56]. Finally, they must possess a suitably high lipid composition with long chain polyunsaturated fatty acids and be free of toxins for target culture species [34,56]. Selection of the suitable microalgal diet is of paramount importance to aquaculture hatchery and nursery success [58]. At present, the most widely cultured species for aquaculture hatcheries and nurseries include *Chaetoceros calcitrans,Isochrysis galbana,Pavlova lutheri,Pseudoisochrysis paradoxa,Tetraselmis suecica* and *Skeletonema costatum.* Other genera include *SpirulinaDunaliellaChlorellaThalassiosiraIsochrysis* and *Nannochloropsis *[49].

## Health benefits of microalgal omega-3 fatty acids

Omega-3 fatty acids represent an important structural component of human cell membranes, particularly neuronal cells [59]. The consumption of EPA and DHA supplements has been shown to prevent cardiovascular, nervous system and inflammatory conditions [60]. With regards to cardiovascular health, regular consumption of ω-3 fatty acids can help reduce the risk of hypertension, thrombosis, myocardial infarction and cardiac arrhythmias [61]. This occurs because ω-3 fatty acids increase the high-density lipoprotein/low-density lipoprotein (HDL/LDL) ratio and decrease the total cholesterol/HDL ratio [61]. In addition to cardiovascular benefits, omega-3 fatty acids have also demonstrated positive effects on brain function and the nervous system [62]. In pregnant women, the adequate intake of EPA and DHA is crucial for healthy development of the fetal brain [63]. In infants, arachidonic acid (ARA), an omega-6 fatty acid, and DHA are also required for normal growth and functional development [64]. Interestingly, increased consumption of DHA may also diminish the severity of depression [65]. Immuno-modulatory effects have been observed when ω-3 fatty acids were used in the treatment of inflammatory conditions such as rheumatoid arthritis, Crohn’s disease, ulcerative colitis, psoriasis, asthma, lupus and cystic fibrosis [66,67]. Children ingesting fish oil more than once a week had a lower probability of suffering from asthma [68]. Increasing the levels of DHA and EPA in patients with rheumatoid arthritis and ulcerative colitis has also been found to reduce pain and improve conditions, although the modes of operation are unclear at this point [69,70].

There is currently a large demand for microalgae in the nutraceutical and pharmaceutical industry due to their health-promoting effects. Microalgal-derived PUFA, such as ARA and DHA are added as fortifications to infant formulae—an industry that is worth $10 billion per annum alone. To date, microalgal extracts can be found in many face and skin care products, e.g. anti-aging cream, refreshing or regenerative care products, sun cream, emollient and anti-irritant in peelers [19]. Dermochlorella is actually extracted from *Chlorella vulgaris,* which can stimulate collagen synthesis in skin supporting tissue regeneration and wrinkle reduction [71]. Protulines is a protein-rich extract from *Arthrospira* (*Spirulina*), which helps combat early skin aging, exerting a tightening effect and preventing wrinkle formation [72].

## Omega-3 fatty acid production in microalgae

Microalgae produce a variety of compounds to help in the adaptation and survival of different environmental conditions. Many marine microalgal strains have oil contents of between 10–50%, (w/w) and produce a high percentage of total lipids (up to 30–70% of dry weight) [1]. The accumulation of fatty acids is closely linked to microalgal growth stages, functioning as an energy stockpile during unfavourable conditions or cell division. Omega-3 is accumulated due to its high energy content, as well as the good flow properties crucial for cellular functions [73,74]. To date, the ω-3 fatty acid content of numerous microalgae strains have been studied. Strains from the genera *Phaeodactylum, Nannochloropsis,Thraustochytrium* and *Schizochytrium* have demonstrated high accumulation of EPA and/or DHA. *Phaeodactylum tricornutum *[38] and *Nannochloropsis* sp. [75] demonstrated an EPA content of up to 39% of total fatty acids, while strains such as *Thraustochytrium *[76] and *Schizochytrium limacinum *[77] contained a DHA percentage of between 30–40% of total fatty acids when grown heterotrophically. High biomass and commercially acceptable EPA and DHA productivities are achieved with microalgae grown in media with optimized carbon and nitrogen concentrations and controlled pH and temperature conditions [78]. High oil production, including DHA from *Schizochytrium* (50% w/w), can be obtained as a result of high growth rate by controlling of nutrients such as glucose, nitrogen, sodium and some other environmental factors, such as oxygen concentrations as well as temperature and pH, achieving high cell densities and DHA productivities [1].

## Induction of omega-3 production in autotrophic microalgae

An increase in microalgal lipid content can be induced by a sudden change of growth conditions. The accumulation of starch and/or lipids reserves is considered a survival mechanism in response to growth-limiting stresses [17], such as UV radiation [79], temperature [80] and shock or nutrient deprivation [81,82], as long as light conditions are present that still allow efficient photosynthesis. For example, during nutritional deprivation (e.g. nitrogen) and under the provision of light, cellular division of many marine or brackish microalgae is put on hold and cells begin to accumulate lipids [83], leading to a 2–3 fold increase in lipid content. Both total lipid and omega-3 fatty acid production can be adjusted by varying growth conditions. The diatom *Phaeodactylum tricornutum* can be induced to increase its lipid level from 81.2 mg/g of culture dry weight to 168.5 mg/g dry weight [38]. Similarly, *Nannochloropsi*s sp. [84] and *Dunaliella* sp. [85] can achieve a total lipid content of up to 47% and 60% of dry ash weight by modifying the light intensity, temperature and salinity levels. Lipid abundance has also been shown to increase due to anaerobic sulphur deprivation [86] or the addition of extra nutrients [87].

Omega-3 fatty acid biosynthesis can be stimulated by a number of environmental stresses, such as low temperature, change of salinity or UV radiation. For example, *Pavlova lutheri* increased its relative EPA content from 20.3 to 30.3 M % when the culture temperature was reduced to 15°C [88]. Similarly, *Phaeodactylum tricornutum* had a higher EPA content when the temperature was shifted from 25°C to 10°C for 12 h [89]. An increase in PUFAs is expected as these fatty acids have good flow properties and would be predominately used in the cell membrane to maintain fluidity during low temperatures. Salinity may also regulate PUFA biosynthesis, although not in a consistent manner. For example, *Crythecodinium cohniiATCC 30556* increased its total DHA content up to 56.9% of total fatty acids when cultured in 9 g/L NaCl. Other treatments that cause the generation of reactive oxygen species and lipid peroxidation also result in higher PUFA contents. For example, *Phaeodactylum tricornutum* increased its EPA content up to 19.84% when stressed with UV light [90]. Some of the increased PUFAs are used to repair membrane damage but as PUFAs contain many double bonds, these also act as an antioxidant by scavenging free radicals.

## Metabolic engineering of microalgae for higher omega-3 contents

**Figure 3:**
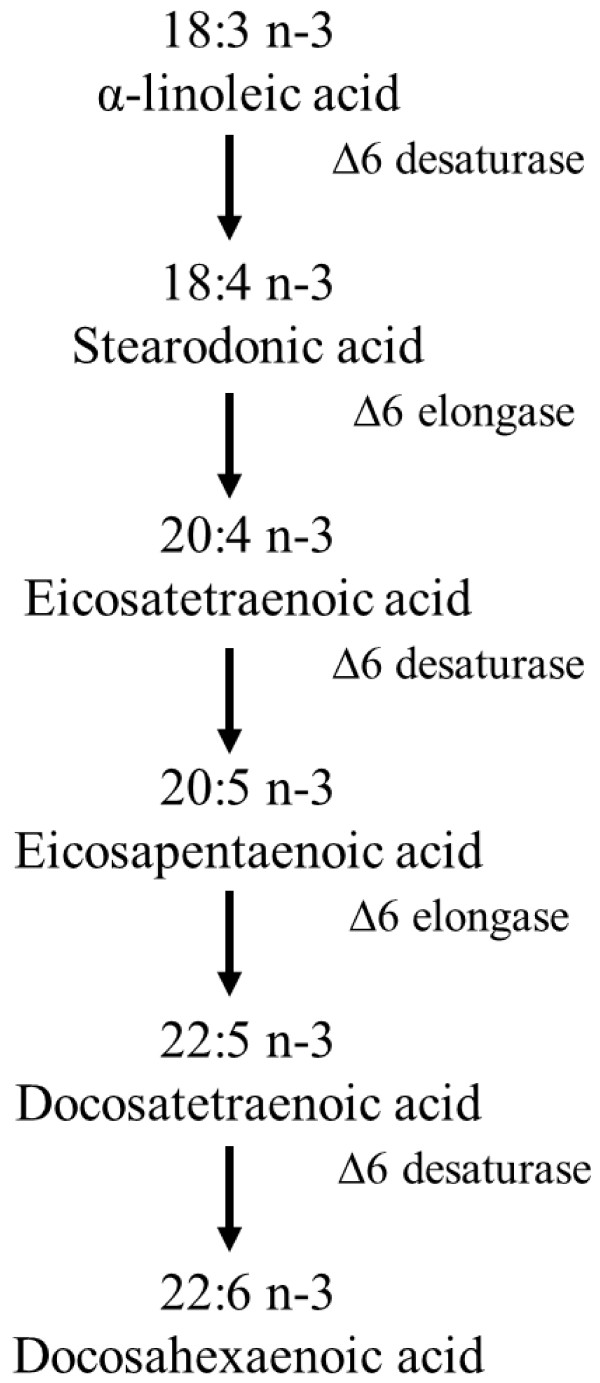
**Conventional Δ6 pathway for biosynthesis of EPA and DHA polyunsaturated fatty acids **[100].

Apart for external stresses, metabolic engineering is another promising approach to increase the production of fatty acids in microalgae (for a recent review see Schuhmann et al. [91]). Genes encoding key enzymes involved in the fatty acid biosynthesis have been identified in *Ostreococcus tauri *[92]*, Thalassiosirapseudonana *[93-95], *Phaeodactylum tricornutum *[96,97] and in particular the model organism *Chlamydomonas reinhardtii *[98]. At present, the mechanisms involved in the fatty acid biosynthetic pathways in microalgae have not been extensively studied and most information has been gathered from studies on plant metabolism. Briefly, *de novo* fatty acid synthesis occurs in the chloroplast and involves the carboxylation and condensation of acetyl-CoA to malonyl-CoA, with further elongation reactions occurring with malonyl ACP as substrate to create long chain fatty acids. Long chain fatty acids are transferred to glycerol-3-phosphate to form triacylglycerol (TAG) via the metabolic intermediate phosphatidic acid in the endoplasmic reticulum [99]. Synthesis of ω-3 fatty acids occurs via the elongation and desaturation of long chain fatty acids (Figure 3).

Work has been performed to create recombinant sources of ω-3 fatty acids in a variety of systems with some success [101,102]. Canola (*Brassica napus*) seeds have been produced which overexpress the *B. napus* Δ15 desaturase, as well as the Δ6 and Δ12 desaturases from the commercially grown fungus *Mortierella alpina* to synthesize the ω-3 fatty acid stearidonic acid (SDA) [14]. It may be possible in the future to produce ω-3 fatty acids in microalgae in much larger quantities by regulating the expression of similar enzymes. A promising cisgenic approach for microalgae maybe to increase EPA or DHA production by overexpressing at least some of their native elongases and desaturases. It may be necessary to use promoters inducible by external stimuli rather than constitutive promoters that may interfere with normal cell function and growth. Another, yet unexplored option may lie in the inhibition of PUFA degradation. β-oxidation of fatty acids occurs in the peroxisomes but before PUFAs can be metabolized, saturases are required to fill in the double bonds. Mutations in one or several saturases may result in less efficient β-oxidation of PUFA and a higher percentage of these fatty acids. However, at present the mechanism behind the selection and storage of fatty acids for triacylglycerol production remains unclear.

## Extraction and purification of omega-3 fatty acids from microalgal biomass

**Figure 4:**
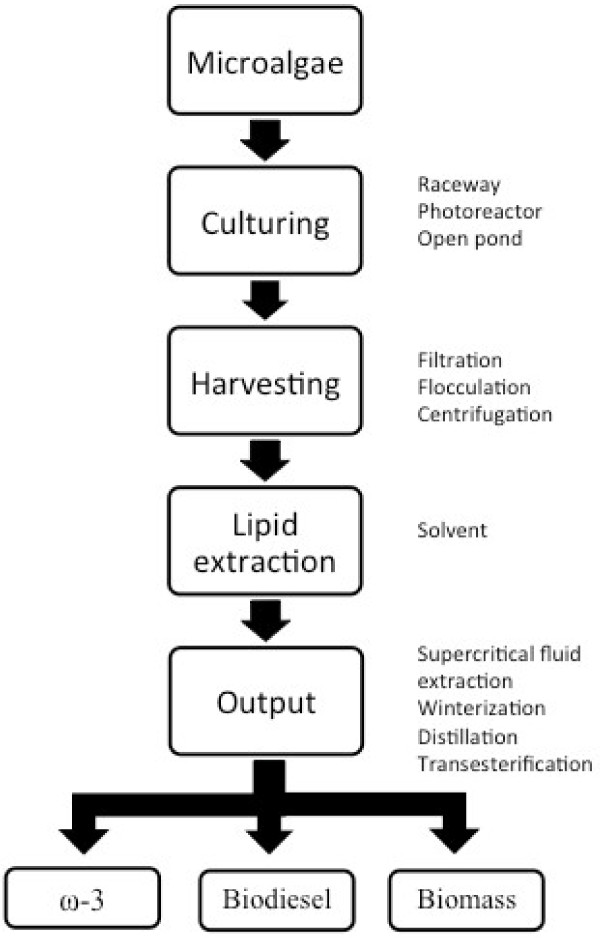
**Examples of a bioprocess production chain in a microalgal biorefinery. **Apart from omega-3 fatty acids (ω-3), the product portfolio includes biodiesel and protein-rich animal feed from the remaining biomass.

Figure 4 summarizes an integrated system for the large-scale production of microalgal bio-products. A microalgae strain is cultivated to increase cell density using photobioreactors, open ponds, race ways or hybrid systems. Algal cells are separated from culture media by filtration, flocculation or centrifugation, followed by drying to improve extraction [1]. Lipid extraction is then commonly performed using a non-water miscible organic solvent. A typical extraction protocol in small scale is often based on the method of Bligh and Dyer [103], which uses a solvent mixtures made of methanol/chloroform for the cell disruption and lipid extraction. Larger scale extraction is typically carried out with hexane as a solvent. Subsequently, unsaturated fatty acids are separated from the total lipids by fractional (molecular) distillation or winterization, whereby oil temperature is reduced to precipitate the more saturated lipids. Further processing to improve the quality, shelf-life and quantity of PUFA oil can include filtration, bleaching, deodorization, polishing and antioxidant addition [1,104] (Table 2).

**Table 2:** Summary of PUFA enrichment processes

**Method**	**Procedure**
Molecular distillation (Fractional distillation)	Purification of fatty acid esters in a vacuum system based on the different boiling points of different fatty acids [105].
Molecular sieves	Separation via membrane permeability and selectivity [106].
PUFA transformations	Esterification of PUFA and free fatty acids to produce esters (ethyl-, glyceryl-, sugar-, other). Inter-esterification to enrich lowly unsaturated fatty acids with PUFA [107].
Super Critical Fluid Extraction	Optimization of lipid solubility and fractionation in supercritical CO_2_[108].
Urea complexation	Solubilization of fatty acids, adding urea and ethanol to saturation point exposing it to heat. Recovery of product by filtration [109].
Winterization	Temperature reduction to render more saturated fats insoluble [110].

Efforts have been made to use lipases, hydrolysis and esterification processes to selectively enrich PUFAs. The main application of lipases on PUFAs is the generation of non-natural esters of these products for use as pharmaceutical products or other synthetic bioactive compounds or their precursors [1]. The effectiveness of harvesting and extraction techniques depends on the microalgal strain's physical characteristics (e.g. cell size and cell wall properties) and the use of the end product. In aquaculture, microalgae are used as a fresh product or as dry pellets which preserve the nutritional content of microalgae [57,58,111]. In this case, microalgal biomass is first de-watered either by filtration, dissolved air flotation, flocculation or sedimentation and then dried to form pellets or directly administrated to livestock [111]. When produced for the pharmaceutical industry, further extraction and purification processes are required. Currently, methods such as supercritical fluid extraction, winterization and fractional (molecular) distillation are used for the extraction and purification of PUFA from microalgae [112,113] (Table 2)

## Omega-3 fatty acid production: a biorefinery approach

The natural capacity of microalgae to produce multiple products, (e.g. oils, proteins and carbohydrates) has encouraged the development of a biorefinery concept for processing. Akin to the petrochemical industry, where crude oil is processed to yield petroleum and a range of other chemicals, microalgae can be processed to produce a range of bioproducts. Different industries are able to use different algal products. For instance, the pharmaceutical and nutraceutical industries use high value bioactive products such as ω-3 fatty acids and carotenoids; the transport industry can use fatty acids from TAG for biodiesel, the chemical industry can use products such as glycerine, while the majority of the biomass can be used by agriculture and aquaculture as animal feed [114,115]. Additional processes that address nutrient recycling and carbon sequestration can be used by anaerobic digestion of wet biomass and pyrolysis for the production of biochar.

Undoubtedly, the biggest interest in microalgal use is for biodiesel production. It potentially represents a more sustainable alternative to fossil fuels as microalgal production facilities do not need to compete for arable land or freshwater. Furthermore, in comparison to land plants, 10–400 times more energy per acre can potentially be produced from microalgae. Although there has been considerable interest and research over the past years into microalgal biofuel production [83], no commercial enterprise has successfully established itself as a supplier of autotrophically derived algal biofuels for any duration. Nevertheless, decreasing fossil fuel reserves and increasing fuel costs continue to drive research targeted towards economically viable production of microalgal biodiesel, with the level of improvement necessary now appearing attainable [15,17]. There is confidence among companies producing microalgae that the production of a high value product, such as omega-3 from microalgae, will further assist in the establishment of the microalgae industry. Several companies have (at least temporarily) shifted their focus from algal biodiesel production, to high value products such as omega-3 and protein-rich biomass as animal feed (e.g. Aurora Algae, MBD, Cellana).

## Conclusions

Global fish stocks are declining and cannot provide a sustainable source of omega-3 fatty acids. Heterotrophic microalgae have been used for the production of omega-3 fatty acids, in particular DHA. However, as the primary producers of PUFAs, the use of autotrophic microalgae for large-scale production of omega-3 fatty acids has recently attracted a lot of interest. Autotrophic microalgae do not require an organic carbon source and hence may avoid the problems faced for heterotrophic cultures that can easily get contaminated with other microorganisms. In a biorefinery concept, omega-3 fatty acids can be separated from microalgal lipids which would be widely used for biodiesel production, while biomass can find uses as valuable protein-rich animal feed which could free up arable land for food production. If carried out at a large scale this would address three major areas of importance: human health, transportable energy and food security.

Over the past decade, algae biotechnology has grown steadily into a global industry with increasing numbers of entrepreneurs attempting to utilize its biochemical diversity for a wide array of applications. At present, achieving economically viable production of microalgal lipids is still a major challenge, but strong potential stems from the fact that these microbial cell factories have not been domesticated and are not as well studied compared to agricultural crops [102]. Indeed, of approximately 40,000 algal species, only a few thousand strains are kept in collections, a few hundred are investigated for chemical content and approximately half a dozen are cultivated in industrial quantities. Therefore, continued isolation and screening of microalgae is required, as well as more in depth studies into algal physiology, biochemistry and genetics. Meanwhile the processes for algae cultivation, harvesting and oil extraction need to be further improved in efficiency and costs. As omega-3 fatty acids are one of the most valuable products from microalgae, they are likely to be the “game-changer” towards large-scale economical microalgae cultivation that will catalyze the production of other important algal bioproducts.

## Competing Interest

The authors declare that they have no competing interests.

## Authors’ contributions

All authors contributed in data collection from literature and writing of the manuscript including figures and tables. All authors have read and approved the final manuscript.
